# Break on through: The role of innate immunity and barrier defence in atopic dermatitis and psoriasis

**DOI:** 10.1002/ski2.99

**Published:** 2022-02-16

**Authors:** H. C. Hawerkamp, C. M. R. Fahy, P. G. Fallon, C. Schwartz

**Affiliations:** ^1^ Trinity Biomedical Sciences Institute, School of Medicine, Trinity College Dublin Dublin Ireland; ^2^ Paediatric Dermatology Children's Health Ireland at Crumlin Dublin Ireland; ^3^ Royal United Hospitals NHS Foundation Trust Bath UK; ^4^ National Children's Research Centre Our Lady's Children's Hospital Dublin Ireland; ^5^ Clinical Medicine Trinity College Dublin Dublin Ireland; ^6^ Mikrobiologisches Institut ‐ Klinische Mikrobiologie, Immunologie und Hygiene Universitätsklinikum Erlangen and Friedrich‐Alexander Universität (FAU) Erlangen‐Nürnberg Erlangen Germany; ^7^ Medical Immunology Campus Erlangen FAU Erlangen‐Nürnberg Erlangen Germany

## Abstract

The human skin can be affected by a multitude of diseases including inflammatory conditions such as atopic dermatitis and psoriasis. Here, we describe how skin barrier integrity and immunity become dysregulated during these two most common inflammatory skin conditions. We summarise recent advances made in the field of the skin innate immune system and its interaction with adaptive immunity. We review gene variants associated with atopic dermatitis and psoriasis that affect innate immune mechanisms and skin barrier integrity. Finally, we discuss how current and future therapies may affect innate immune responses and skin barrier integrity in a generalized or more targeted approach in order to ameliorate disease in patients.

## Graphical abstract

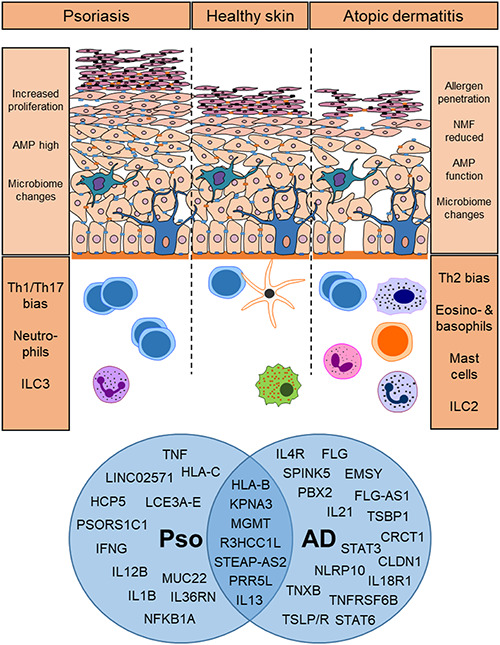

## 1 INTRODUCTION

### 1.1 The skin barrier in healthy human skin

The human skin is the largest and one of the most important immunologically active organs.^1^, ^2^ Due to its location as the outer surface of the body, the skin must be able to protect the body against all types of environmental threats.

***Structure—*** The skin can roughly be divided into three layers: the epidermis—the outermost layer—consisting mainly of keratinocytes in various differentiation states, the dermis, where blood and lymphatic vessels are found and the majority of immune cells reside, and the inner layer containing the subcutaneous fat.^3^, ^4^

***Physical Barrier function—*** Keratinocytes form the epidermal skin layer and initially protect from threats, such as UV irradiation, pathogen/allergen entry or water loss. Starting from the inner epidermal layer, one finds rapidly proliferating, undifferentiated keratinocytes. These basal keratinocytes play an important role in the production of proteins and lipids, and later differentiate to form the stratum spinosum, change their shape and proliferate further.^3^ Reaching the stratum granulosum, the keratinocytes are at their maximum production of lipids and proteins. Keratinocytes progress through a terminal differentiation programme to form the stratum corneum. The outermost layers of the stratum corneum consist of keratinocytes that are reduced to anucleate cells without organelles (corneocytes) and then shed through enzymatically controlled desquamation.^3^, ^4^, ^5^ Here, corneocytes play a central role in the skin barrier as they prevent external substances such as pathogens and allergens from entering, and water from leaving the skin, thus preventing water loss (xerosis).^3^ A key molecule providing structure and integrity here, is the filament‐aggregating protein (filaggrin), a loss of which enhances inflammatory skin conditions, such as atopic dermatitis (AD).^6^, ^7^ The importance of skin integrity in the pathogenesis of AD was emphasised by the association of loss‐of‐function mutations in the filaggrin gene (*FLG*) with AD.^8^ This work indicated that epidermal barrier dysfunction is a primary aetiological phenomenon in AD rather than a consequence of disrupted immunology.^8^ A meta‐analysis of studies on *FLG* mutations and atopic dermatitis risk showed evidence that *FLG* mutations have the strongest association with risk of atopic dermatitis due to skin barrier deficiency in genetic variants that have been investigated thus far.^9^

The skin‐intrinsic defence against pathogenic microbes is enhanced by the production of antimicrobial peptides (AMPs) by keratinocytes in the deeper layer of the epidermis.^10^ AMPs such as LL‐37, human beta‐defensins (hBD) or S100 proteins show a broad antimicrobial activity against bacteria and fungi. Interestingly, interleukin (IL)‐26 produced by subsets of T helper (T_H_) cells also shows antimicrobial features—while being upregulated in psoriasis—and is thus bridging innate and adaptive immunity.^11^, ^12^ Interestingly, a recent study indicates that self‐DNA is released in AD skin lesions, which then binds to AMPs and reduces anti‐microbial activity.^13^ Thus, AMPs are functionally altered during AD and psoriasis.

### 1.2 Innate immune mechanisms in the skin

The skin is populated with a complex array of blood‐derived and tissue‐specific immune cells. Several immune cell populations reside in the epidermis: primarily Langerhans cells (LC; specialised epidermal dendritic cells [DC]), monocyte‐derived LC‐like cells and inflammatory dendritic epidermal cells (IDECs),^14^ but also tissue‐resident CD8^+^ T cells. LCs are among the first line of defence, acting as immune sentinels, which migrate to skin‐draining lymph nodes upon pathogen encounter.^15^ While LCs and LC‐like cells are found in the skin at healthy steady state, their numbers strongly increase in inflammatory conditions whereas IDECs only populate the skin under inflammatory circumstances. The majority of immune cells are found in the dermis, where—similar to the epidermis—the cell numbers are very low at steady state but show a strong surge in inflammation. The specialised immune cells here are plasmacytoid DCs, dermal DCs, tissue macrophages, different subtypes of CD4^+^ T cells, such as T helper type 1 (T_H_1) cells, T_H_2 cells, and regulatory T cells (T_reg_), but also natural killer (NK) T cells and different types of innate lymphoid cells (ILC).^4^ With regard to their transcription factors and cytokine production, type 1, 2 and 3 ILCs closely resemble the three major T_H_1, T_H_2 and T_H_17 cell subpopulations, respectively. Importantly, ILCs do not require traditional adaptive immune receptor rearrangement and can thus react to innate signals without antigen‐specificity.^16^ Similar to classical T_H_ cells, an imbalance of cutaneous ILCs enhances inflammatory skin disease manifestations.^17^, ^18^ ILC1 are the least well characterised ILC subset and their role in skin inflammation remains elusive. Based on their cytokine profile it is hypothesised that they are involved in allergic contact dermatitis.^19^ ILC2 produce the cytokines IL‐5 and IL‐13 and have been implicated in AD, where they were found to be highly enriched in lesional skin.^20^ In line with the T_H_17‐resembling cytokine profile, ILC3 are increased in blood and skin of psoriasis patients.^21^ More evidence for their implication in the pathogenesis of psoriasis stems from the observation that mice that lack adaptive lymphocytes still develop psoriasiform inflammation similar to wild‐type controls that possess T_H_17 cells.^22^

Although mostly considered of structural importance to the skin, keratinocytes play their part in the resident skin (innate) immune system. They contribute to immune surveillance by expressing a range of toll‐like receptors (TLRs; TLR1‐6 and 9) and producing AMPs like LL37.^23^ Most importantly, keratinocytes are capable of producing a wide range of chemokines and cytokines like CXCL8,^24^ chemokine (C‐C motif) ligand 20 (CCL20)^25^ and IL‐23.^26^ These chemokines and cytokines can in turn attract immune cell and their regulation is key to maintain a homoeostasis in healthy skin.

### 1.3 Innate immune mechanisms in Cutaneous diseases

#### 1.3.1 Psoriasis

Psoriasis is a common chronic inflammatory skin disease spanning a variety of skin phenotypes and is linked to complex comorbidities including seronegative arthritis, ischaemic heart disease, and metabolic syndrome.^27^, ^28^, ^29^ Histologically, a strikingly thickened epidermis is found, together with deep epidermal ridges reaching into the dermis.^27^ In psoriatic lesions, T_H_1 and T_H_17 cells are predominantly found together with an increased expression of IL‐17 and IL‐22.^30^ A key player accountable for the thickened epidermis is IL‐22, which promotes proliferation of keratinocytes.^31^, ^32^, ^33^ Additionally, an increased amount of IL‐23 and IL‐1β produced by DCs drives T_H_17 cell differentiation and thereby accelerates the progression of the psoriatic phenotype.^34^ While T cells and especially T_H_17 cells have been seen the crucial culprits in the pathogenesis of psoriasis, an increasing body of evidence shows the role of the innate immune system.^23^, ^35^ Reports hint towards more influence of the adaptive immune system in mild psoriasis, whereas severe psoriasis is more influenced by actions of the innate immune system.^36^ An increasing role in the pathogenesis of the disease has been attributed to ILC3 cells which are found increased in psoriasis.^21^, ^37^ Due to their ability to produce T_H_17‐cytokines such as IL‐17 and IL‐22, they bridge adaptive and innate immunity. A recent study has shown that quiescent‐like ILC2 in the skin can transition into pathogenic ILC3‐like cells upon disease initiation.^38^ ILC3 numbers in psoriatic skin are reduced after therapeutic treatment with anti‐tumour necrosis factor (TNF) antibody, indicating their contribution to pathogensis.^21^ It has further been described that in psoriasis, epidermal LC have impaired migratory capacity to skin‐draining lymph nodes and thus delayed onset of cutaneous immune responses.^39^ Additionally, the capacity of the structural keratinocytes to produce a variety of chemokines and cytokines together with the hyperproliferation seen in psoriasis leads to a vicious cycle in the pathogenesis of the disease.^23^ The strong upregulation of chemokine ligand CCL20 expression in keratinocytes in presence of T_H_17‐derived IL‐17A leads to further recruitment of T cells into psoriatic lesions.^25^ Additionally, IL‐17 is stimulating the proliferation of keratinocytes and their secretion of AMPs thereby contributing to the hyperproliferation phenotype.^40^ While the general action of IL‐17 in the pathogenesis of psoriasis is known, further research is necessary to elucidate cell‐specific contributions such as the role of ILC3‐derived IL‐17.

#### 1.3.2 Atopic dermatitis

AD, also known as atopic eczema, is a chronic inflammatory skin disorder characterised by severe pruritus, dry and scaly skin, as well as raised, red lesions in the bends of arms and legs.^41^, ^42^, ^43^, ^44^ The prevalence is approx. 10%–20% in developed countries and in approximately 60% of the cases, onset of disease is in the first year of life.^45^ In contrast to psoriasis, AD is a disease with a type 2‐biased phenotype with increased expression of IL‐13 and IL‐5.^46^ Chronic AD, however, also displays IFN‐γ, the signature cytokine for T_H_1 cells and type 1 responses.^47^, ^48^ In line with T_H_2 cells, ILC2s have also been reported, to be highly enriched in lesional AD skin.^49^, ^50^, ^51^ Skin ILC2 do not rely on IL‐33 signalling but instead on thymic stromal lymphopoietin (TSLP).^20^ TSLP is strongly increased in AD, leads to the production of the T_H_2 cell attracting chemokine CCL17,^52^ and considered to be a trigger factor in the initial stages of the disease.^53^ Furthermore, TSLP has been reported to stimulate cutaneous neurons to promote itch and provoke itching.^54^ The scratching in response to itching sensation will break down skin barrier functions leaving AD patients largely unarmed against skin infections. It is therefore not suprising that up to 90% of AD patients are colonised with *Staphylococcus aureus* and are also prone to viral infections caused by herpes simplex virus.^43^ ^,^ ^55^, ^56^, ^57^ Although AD patients with higher *S. aureus* abundance show significantly higher excoriations and sleep loss, a correlation between (patient reported) itch intensity and *S. aureus* concentration is not evident.^58^ A reason for this might be the lower levels of AMPs (e.g. LL37 and hBD2) in AD compared to psoriasis.^59^, ^60^ This reduced AMP expression by keratinocytes is partly caused by the inhibitory effects of the T_H_2 cyokines IL‐4 and IL‐13,^61^ as well as IL‐10^62^ and TSLP.^63^

## 2 MODULATION OF SKIN BARRIER FUNCTION

### 2.1 Single nucleotide polymorphisms (SNPs) affecting skin barrier integrity and innate immunity in AD and psoriasis

**FIGURE 1:**
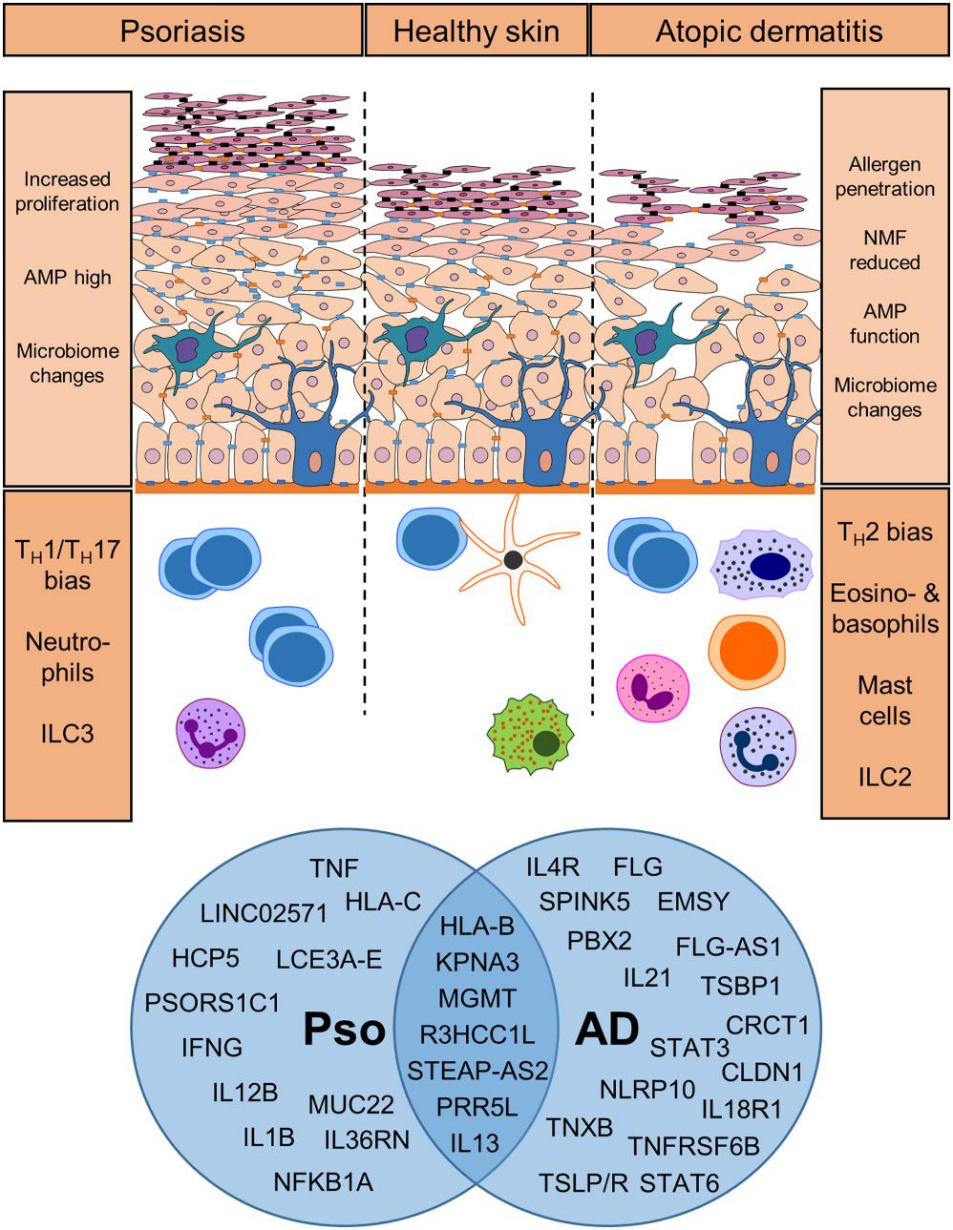
**Schematic representation of skin and underlying processes during steady state, psoriasis and atopic dermatitis**. In psoriatic lesions increased cell proliferation occurs in the epidermis and elevated production of anti‐microbial peptides (AMPs), while during AD the skin barrier is impaired, which leads to increased allergen penetration, reduced natural moisturising factor (NMF) and may affect AMP abundance or activity. Both diseases are characterised by changes in microbiome composition. During psoriasis pro‐inflammatory T_H_1 and T_H_17 cells dominate the affected skin. In contrast, type 2‐associated cells, including T_H_2 cells, eosinophils, basophils and ILC2 can increase during AD. GWAS analysis (publicly available databases: GWAS database, https://www.ebi.ac.uk/gwas/; GWAS Central, https://www.gwascentral.org/; accessed 15.11.2021; filtered for psoriasis and atopic eczema traits) of the most common SNPs associated with psoriasis (Pso) and atopic dermatitis (AD) are summarised in the Venn diagram

Both AD and psoriasis are multifactorial diseases with a complex origin. Comparison of SNPs or genetic variants, between healthy controls and persons suffering from AD or psoriasis revealed distinct sets of mutations associated with either disease^64^ (Figure 1). These genome‐wide association studies lay the groundwork for the design of novel therapeutic options as they help to dissect the molecules and mechanisms involved in pathogenesis. Relevant genetic variants associated with AD span genes—and their products—involved in the initiation of immune responses, effector cytokines and chemokines, signalling molecules, and, importantly, proteins involved in the maintenance of skin barrier integrity. Important genes and the disease‐associated genetic variants are listed in Table 1. Detailed reviews on the genetics of AD and psoriasis were also recently published by Martin et al.^65^ and Ogawa and Okada,^66^ respectively.

**TABLE 1:** Selected SNPs associated with AD and/or psoriasis

Gene	Reference SNP cluster ID	Reference
Atopic dermatitis		
*IL1RL1*	rs3917265, rs1861246, rs13015714, ‐26999A	[67, 172, 173, 174]
*TSLP/R*	rs111267073, rs10043985, rs2289276, rs1898671, rs11466749, rs2416259, rs1837253, rs3806932, rs2289278, rs36139698, rs36177645, rs36133495	[68, 175, 176, 177, 178, 179]
*IL18* *R*	rs13015714, rs6419573, rs13015714, rs1861246	[172, 173]
*IL21*	rs17389644	[64, 172, 180]
*IL6R*	rs2228145, rs12126142, rs4576655, rs12730935	[172, 173, 181]
*IL4/R*	rs2107357, rs143021546, ‐590 C/T	[88, 176, 182]
*STAT6*	rs1059513, rs3024971	[175, 183]
*STAT3*	rs4796793, rs17881320, rs12951971	[172, 184, 185]
*FLG*	rs558269137, rs6661961, rs3126085, 2282del4, rs61816761, rs150597413, rs138726443	[65, 89, 173, 175, 181]
*TMEM79*	rs6694514	[98]
*EMSY*	rs7130588, rs2212434, rs7110818, rs7927894, rs2155219, rs34455012	[64, 89, 172, 173, 180, 181, 182, 186]
*CLDN1*	rs893051	[187]
*LCE3E*	rs10888499, rs61813875	[64, 172]
*LCE5A*	rs6661961, rs11205006, rs471144, rs12144049, rs12081541	[64, 89, 184, 188]
*SPINK5*	rs2303063, rs2303067	[189, 190]
Psoriasis		
*PSORS1* region (including *HLA‐C*)	rs6913137, rs2853950, rs3130573, rs7756521, rs9263717, rs2233959, rs2524096, rs12199223, rs1265078, rs2249742, rs2853961, and many more	[191, 192]
*TNFAIP3*	rs582757, rs610604, rs6933987, rs643177	[181, 193, 194, 195, 196]
*NFKB1A*	rs8016947	[193, 196]
*IL36RN*	rs387906914, rs397514629	[75, 197, 198]
*LCE3B*	rs4845454, rs11205044, rs1581803	[64, 199, 200]
*LCE3D*	rs4085613, rs4112788, rs6677595	[193, 194, 201]
AD and psoriasis		
*HLA‐A/B*	AD: rs148203517, rs4713555, rs28752924, rs28383323, rs200291258, rs9405068, rs4713555, rs2251396; Pso: rs4406273, rs2523619, rs17728338, rs75851973, rs76956521, rs1960278, rs12212594, rs4959062, rs4349859, rs10484554	[64, 80, 172, 173, 181, 191, 193, 196, 202]
*CARD14*	AD: rs535171797; Pso: rs11652075	[193, 203]
*IL12B*	AD: rs3212227, rs393548, rs436857; Pso: rs2082412, rs3212227, rs3213094, rs2546890, rs12188300, rs12188300, rs6887695	[78, 193, 194, 195, 204, 205, 206]
*IL13*	AD: rs848, rs1295686, rs847, rs1295685, rs20541, rs12188917; Pso: rs20541, rs1295685, rs847	[64, 89, 172, 173, 175, 180, 181, 193, 196, 199, 207]
*MGMT*	rs80312298	[64]
*PRR5L*	rs2592555, rs12295535, rs11033603, rs2218565	[64, 172, 173, 180]
*KPNA3*	rs3736830	[64]
*R3HCC1L*	rs11189494	[64]
*STEAP‐AS2*	rs7798970	[64]
*PELI2*	rs17761563	[64]

The keratinocyte‐derived so‐called ‘alarmin cytokines’ are important inducers of skin immune responses. The −26999 G/A mutation in the distal promoter of *IL1RL1* (the receptor for IL‐33; ST2) leads to the increased expression of soluble ST2 (IL‐33 decoy receptor), preferential activation of T_H_2 cells, high total IgE, and an increased odds ratio (1.87) to develop AD.^67^ SNPs in *TSLP* and its receptor (*TSLPR)*, have also been correlated to the development of AD.^68^ Studies in animal models have confirmed a pathogenic role for TSLP, as its overexpression in keratinocytes leads to the development of AD‐like inflammation. SNPs in *IL18* are also associated with AD.^69^, ^70^ In psoriasis, IL‐18 is produced by activated keratinocytes and can act on DC and promote type‐1 and type‐3 responses. In AD, IL‐18 can act in concert with IL‐12 to drive type‐1 responses or promote type‐2 responses together with TSLP. Recently, it was found that IL‐18 can activate ILC2, which may contribute to skin inflammation through their production of IL‐13.^71^ These SNPs highlight the importance of molecules that signal the presence of potentially harmful events as they are generally released upon tissue damage. The functional characterisation of IL‐25, IL‐33 and TSLP in the outset of the cutaneous inflammation has led to the development of biologics targeting each cytokine or their cognate receptor for AD and indeed other allergic disorders.

In psoriasis, alarmins do not seem to play such a prominent role in the initiation phase of the immune response. Instead, human leucocyte antigen (HLA)‐related gene variants correlate with an increased odds ratio to develop psoriasis.^72^ The *PSORS1* locus maps to the major histocompatibility complex (MHC) region and spans nine genes. For example, a variant of HLA‐C (HLA‐Cw6)^73^ can—in addition to conventional antigens—present autoantigens to CD8 T cells. Activation of pro‐inflammatory pathways is also involved in the pathogenesis of psoriasis, including *TNFAIP3*, *NFKBIA* and *CARD14*.^74^ Here, the immunoregulatory or inhibitory functions of TNFAIP3 and IkB are decreased and promote pro‐inflammatory NFkB activation. Similarly, gain‐of‐function mutations in *CARD14* increase NFkB activation. Mutations in the gene encoding for IL‐36 receptor antagonist (*IL36RN*) are associated with the development of generalised pustular psoriasis, raising potential for targeting IL‐36—a member of the IL‐1 family—in psoriasis.^75^ Indeed, targeted deletion of IL‐36R on keratinocytes led to decreased expression of pro‐inflammatory cytokines.^76^

Downstream of the initiation of an immune response, SNPs in the signalling cascade of cytokines—mainly the respective JAK‐STAT pathway—are associated with AD. Here, the divergent nature of AD (type‐2‐biased) and psoriasis (type‐1/3‐biased) becomes apparent. IL‐12B is a subunit of the cytokine IL‐12 that promotes the differentiation of T_H_1 cells. Genetic variations in *IL12B* are associated with psoriasis in a cohort of Danish patients,^77^ while SNPs in *IL12B* decrease the risk of developing AD.^78^ Similarly, IL‐21 can drive T_H_17 differentiation in psoriasis,^79^ with higher levels of IL‐21 reported in lesional skin of psoriatic patients. The genomic region (4q27) including the *IL21* gene is associated with psoriasis and SNPs in this locus are associated with higher IL‐21 levels in other inflammatory disorders.^80^ In addition to T_H_17 and T_H_1 activation, IL‐21 can cause epidermal hyperplasia. While increased levels of IL‐21 are found in acute lesions of AD patients,^81^ the implication for AD pathogenesis requires further study. In mouse models of AD‐like inflammation, lower levels of the IL‐17‐induced T_H_2‐recruiting chemokine CCL17 have been observed,^82^ and a protective role for the IL‐17RA in AD has been described.^83^ Certain SNPs in *IL6*—required for balancing T_H_17/T_REG_ cells^84^—are associated with a decreased risk to develop psoriasis,^85^ while it may increase the risk for AD^86^ and disruption of IL‐6‐receptor signalling improved AD.^87^

Opposed to the pro‐inflammatory type‐1 and type‐3 responses, IL‐4 is the main cytokine driving T_H_2 polarization and is associated with allergic diseases including AD.^88^ IL‐13, another hallmark cytokine of type 2 immune responses, is also associated with AD.^89^ In contrast, IL‐4 is not directly associated with psoriasis but repeated IL‐4 application in psoriatic patients can divert the pro‐inflammatory type‐1/‐3 response towards type‐2.^90^ Both IL‐4 and IL‐13 signal through STAT6, which is also linked to AD pathogenesis,^91^ and mice that express a constitutively active form of STAT6 were shown to develop AD‐like lesions.^92^

Genes involved in the skin barrier are linked to AD and psoriasis. In the *PSORS4* region, more than 60 genes that control keratinocyte differentiation are encoded.^93^ Deletion of *LCE3B* and *LCE3C* (late cornified envelope proteins) is associated with psoriasis.^94^ Importantly, variants of genes involved in the maintenance of the skin barrier are strongly associated with AD. Among the most important affected genes are *FLG* (encoding for filaggrin), *TMEM79* (mattrin), and *EMSY*. First described in 2006, filaggrin is one of the most prominent proteins, in which a SNP can cause ichthyosis vulgaris^95^ and predispose for AD.^8^ To date, more than 50 mutations in *FLG* in all human populations studied around the globe (>1% allele frequency) are reported, suggesting an evolutionary benefit of a mildly leaky barrier. Loss‐of‐function of filaggrin leads to increased abnormal structure and physiology of the skin, increased trans‐epidermal water loss (TEWL) and dry skin, as natural moisturising factor (NMF) is a catabolic product of filaggrin.^96^ Similarly, *Tmem79* mutations were found to increase barrier leakage and AD‐like inflammation in mice.^97^, ^98^ Recently, a possible function of EMSY in maintaining the skin barrier was described. Using skin organoids, it was found that EMSY acts as a transcriptional regulator in keratinocytes.^99^ In skin biopsies of AD patients, EMSY located in the nucleus, actively repressing gene expression.^99^ Furthermore, De Benedetto et al. showed that genetic variants in the tight junction gene CLDN1 are associated with AD.^100^ The strong associations of the described SNPs with AD and psoriasis highlights the necessity of an intact barrier to maintain tissue homoeostasis. Despite the recent advances in the field of 3D‐organ cultures, skin‐on‐a‐chip models and in silico‐prediction models, functional studies on the identified loci are required to work towards novel therapeutics. Thus, animal models of skin disease and clinical studies are still essential for scientific progress.

### 2.2 Keratinocytes recruit immune cells

Keratinocytes are important central modulators of immune responses in the skin. They are not only capable of producing AMPs that act as a first line of defence but can also release chemotactic factors. In psoriasis, keratinocytes release chemokines CXCL9, ‐10, ‐11 and ‐20, as well as CXCL‐1 and ‐8, attracting LC and neutrophils to the skin, respectively. Keratinocytes are also able to sense microbial patterns via TLRs. As a consequence of TLR signalling, keratinocytes can release pro‐inflammatory cytokines, such as IL‐1β and IL‐18, both of which are cleaved via the NLRP‐inflammasome into the active form. IL‐1β can upregulate ICAM‐1 on dermal endothelial cells and may facilitate entry of leukocytes into the skin.^101^ In AD, keratinocytes synthesise increased amounts of chemokines, such as CSF‐2, RANTES/CCL5 and MCP‐1/CCL2, promoting the infiltration of eosinophils, dendritic cells and monocytes, as well as T cells into the skin.^102^ In order to counteract the loss of the skin's barrier function, the stratum corneum increases in thickness. Hyperkeratosis is a hallmark of chronic AD and palmoplantar hyperkeratotic psoriasis and develops from an imbalance of protease–protease inhibitors interactions.^103^ While kallikrein‐(KLK)7 protein levels were increased during AD, its activity was not elevated. Instead the increased levels of lymphoepithelial Kazal‐type‐related inhibitor (LEKTI, encoded by *SPINK5*) were thought to prevent corneodesosome degradation by KLK7. Similarly, unregulated KLK5 activity in the absence of LEKTI led to the development of AD‐like inflammation in mice.^104^ Thus, regulation of proteolytic pathways contribute to skin barrier function and pathogenesis of inflammatory skin disease.

### 2.3 Innate cells can regulate skin barrier function

Innate immune cells in the skin are the first responders to tissue damage and invading pathogens. Innate cells are potent producers of an array of cytokines. The predominant cytokines linked to skin diseases are IL‐17, IL‐4, and IL‐13. IL‐17A can be produced by γδ T cells, iNKT and ILC3, and IL‐17 can activate the release of AMP.^105^ Keratinocytes are a major source of AMPs.^106^ Defective function of these AMPs, such as cathelicidin or β‐defensins may contribute to AD.^107^ Interestingly, decreased AMP production is associated with predisposition to AD, while high AMP expression is observed in psoriatic lesions.^61^, ^108^ LL‐37, a member of the cathelicidin family, which was increased during atopic eczema,^109^ has an essential role in angiogenesis and wound healing^110^ and is released in response to injury.^111^

IL‐4 and IL‐13 are produced by both innate and adaptive immune cells. For example, basophils can release large amounts of IL‐4 upon engagement of the Fcε receptor by IgE crosslinking. Eosinophils are also a significant source of IL‐4 and can be activated by IL‐33 or CSF‐2 as well as TLR and interferons. ILC2 can produce large amounts of IL‐13 and IL‐5, but also—depending on the context—IL‐4. IL‐4 has been shown to massively alter barrier function.^92^ In IL‐4‐deficient animals, expression of skin barrier proteins, such as loricrin, involucrin and transglutaminase‐3, was two‐ to three‐fold increased. Importantly, IL‐4‐deficiency also increased filaggrin expression in the skin. Indeed, keratinocytes decrease expression of filaggrin, loricrin, involucrin, and hornerin in response to IL‐4 and IL‐13, while the peptidase KLK7 was induced. Thus, IL‐4 leads to skin desquamation through degradation of corneodesmosomal proteins. In a recent study, basophil‐derived IL‐4 was also shown to reduce IL‐1 and IL‐23 production from keratinocytes that impaired γδ T cell activation and thus promoted *S. aureus* colonisation.^112^ IL‐13 signals also through the IL‐4Rα chain (heterodimeric receptor with IL‐13Rα1) and probably exerts similar functions, although they are still under debate.^113^ In addition, the second receptor, IL‐13Rα2, functions as a decoy receptor scavenging IL‐13 ameliorating skin barrier defects and cutaneous inflammation.^114^ However, it was also recently shown that homoeostatic IL‐13 from ILC2 in healthy skin—while fostering a noninflammatory skin environment – may predispose for allergic sensitisation through the activation of T_H_2‐priming dermal DC2 subsets.^115^ Interestingly, IL‐33 was shown to disturb skin barrier integrity independently of mast cell and T_H_2‐cell‐derived cytokines.^116^ Whether IL‐33 directly modulates keratinocyte‐function, which increase expression of ST2 in AD lesions^117^ or via the activation of ILC2^118^ remains to be determined.

Pruritus is a major cause for the breakdown of the skin barrier and the crosstalk of innate immune cells with the nervous system is an emerging field. The alarmins TSLP and IL‐33 can induce itch by acting directly on sensory neurons,^54^ activation of ILC2 and basophils,^119^ IL‐31 release from T cells, reducing skin barrier protein expression,^116^, ^120^ or histamine release from mast cells.^121^ Histamine and serotonin release from mast cells activated by IgE‐crosslinking of the FcE‐receptor causes histaminergic itch. Recently, it was discovered that mast cells are also activated by PAMP9‐20, a peptide released by various cell populations, including keratinocytes, acting on Mrgprb2 in mice or MRGPRX2 in humans. Mast cells then only secrete small amounts of histamine and serotonin but instead release tryptase and thereby trigger non‐histaminergic itch.^122^ The release of IL‐4 and IL‐13 by mast cells, eosinophils and basophils may also directly contribute to scratching behaviour as it has been demonstrated that IL‐4 injection induces scratching via signalling through IL‐4Ra and JAK1.^123^ Confirmation of IL‐4Ra‐JAK pathways mediating itch was provided with Dupilumab^124^ and JAK inhibitors^125^ both improving pruritus in treatment of AD for patients.^126^

Taken together, the release of cytokines by immune cells during psoriasis and AD alters both the composition and function of the skin barrier and further may aggravate disease through neuronal circuits. Targeting cytokines and the signalling pathways of the skin neuro‐inflammatory network with monoclonal antibodies and inhibitors have become promising areas of research to develop novel therapies.

## 3 EFFECTS OF THERAPEUTIC APPROACHES ON THE INNATE IMMUNE SYSTEM

In this section, we will highlight novel developments in the treatment of AD and psoriasis that affect mechanisms related to innate immunity and barrier function of the skin.

### 3.1 Topical and systemic therapies

***Emollients and topical barrier treatments*** are used in AD and psoriasis to maintain the skin barrier function, combined with avoidance of detergents. Improvement of the barrier in skin barrier deficiency seen in AD, via disease‐specific, barrier corrective topical treatments such as ceramide‐dominant mixtures with barrier lipids, down‐regulates pro‐inflammatory signalling mechanisms involved in barrier repair. The ingress of further haptens, which drive T_H_2‐type responses are increasingly blocked by skin barrier improvement. A lipid mixture gives an acidic pH on the skin surface adding to the barrier function and blocking the activation of proinflammatory serine proteases.^10^ Itch is a feature of some dermatoses that are associated with skin barrier deficiency. The inclusion of anti‐pruritic ingredients in emollients can supplement the barrier restorative factors of the emollient by decreasing itch, which is a factor in skin barrier deficiency.^103^, ^127^

***Topical corticosteroids (TCS)*** accompanied by emollients have been the mainstay of the treatment of AD since their introduction in the 1950s.^128^ The anti‐inflammatory effect of TCS is mediated through a cytoplasmic glucocorticoid receptor (GCR) in target cells.^42^ One feature of how innate immunity influences anti‐inflammatory effects with TCS is after ligand binding, when the corticosteroid/GCR complex translocates to the nucleus. Various transcription factors including nuclear factor κB (NF–κB), inhibit the transcriptional activity of genes encoding cytokines such as IL‐1, IL‐4, IL‐5, IL‐13, TNF as well as chemotactic proteins and adhesion molecules.^42^ While this immunosuppression limits skin inflammatory processes, TCS therapy is associated with skin atrophy and leads to an impaired skin barrier. Thus, emollients are used in combination to promote barrier restoration.

A similar mode of action is observed when treating psoriasis with TCS. Immune cells and pro‐inflammatory cytokines are repressed by signalling via the GCR. Corticosteroids commonly repress maturation and differentiation of DC and macrophages, thereby further reducing pro‐inflammatory type‐1 and type‐3 responses. In addition, TCS has antimitotic properties reducing the hyperproliferation but also lead to skin atrophy. The impaired skin barrier facilitates TCS penetration and promotes systemic adverse events. Additional treatment with Vitamin D analogues, which corrects epidermal hyperproliferation and induces apoptosis in inflammatory cells, can prevent some of the adverse effects of TCS.^129^ Vitamin D affects the production of AMPs, which are involved in maintenance of the skin barrier, decreased levels of which can results in exacerbations of AD, particulary infective flares of inflammation.^105^ ^,^ ^130^

***Topical calcineurin inhibitors (TCIs),*** such as tacrolimus or pimecrolimus, act as steroid‐sparing agents.^131^ They inhibit inflammatory cytokine transcription in activated T cells and other inflammatory cells through inhibition of calcineurin.^131^ With this anti‐inflammatory activity, topical calcineurin inhibitors help to allow the skin barrier to be restored.^106^, ^132^ Topical calcineurin inhibitors do have the potential for local immunosuppression, however, clinical trials have shown no increase in systemic or local skin infections. Yet, TCI treatment impairs skin barrier function through decreasing epidermal lipid synthesis, suppression of IL‐1α and reducing AMP synthesis.^136^ While TCIs are approved in the treatment of mild‐to‐moderate AD, they show limited efficacy in psoriasis.^137^ Psoriasis that affects facial skin or genital skin can show improvement with the use of TCIs.

***Traditional systemic therapies:*** Despite the usual success of topical therapies at gaining control of a patient's AD, there is a minority of patients for whom systemic therapy is a necessary next step in AD management.^128^ Systemic medications include azathioprine, methotrexate, cyclosporine as discussed in Table 2, which act as steroid‐sparing immunosuppressants,^138^, ^139^, ^140^ which are also indicated in moderate‐to‐severe psoriasis.

**TABLE 2:** Advanced therapeutics for atopic dermatitis and psoriasis

	Compound proprietary name	Target	Details	References
Atopic dermatitis	Dupilumab	IL‐4Rα	IL‐13/IL‐4 inhibitor, monoclonal antibody	[124, 148, 208]
	Topical crisaborale	Phosphodiesterase 4 (PDE4) enzymes	Phosphodiesterase 4 inhibitor, small molecules	[144, 145, 146, 209]
	Oral apremilast			
	Tofacitinib	JAK/STAT pathway	JAK inhibitors, small molecules	[161, 162]
	Baricitinib			
	Upadacitinib			
	Ruxolitinib			
	Nemolizumab	IL‐31Rα	IL‐31 inhibitor, monoclonal antibody	[210]
	Tralokinumab	IL‐13	IL‐13 inhibitor, monoclonal antibody	[150]
	Lebrikizumab			
	Tezepelumab	TSLP	TSLP inhibitor, monoclonal antibody	[143]
	Etokimab	IL‐33	IL‐33 inhibitor, monoclonal antibody	[159]
	Fezakinumab	IL‐22	IL‐22 inhibitor, monoclonal antibody	[211, 212]
Psoriasis	Adalimumab	TNFα	TNF inhibitor, monoclonal antibody	[213, 214, 215, 216]
	Certolizumab pegol			
	Golimumab			
	Infliximab			
	Etanercept	TNFα	TNF inhibitor, fusion protein decoy receptor	[217]
	Brodalumab	IL‐17A	IL‐17A inhibitor, monoclonal antibody	[218, 219, 220, 221, 222, 223]
	Ixekinumab			
	Secukinumab			
	Guselkumab	IL‐23	IL‐23 inhibitor, monoclonal antibody	[224, 225, 226]
	Risankizumab			
	Tildrakizumab			
	Ustekinumab	IL‐12/IL‐23	IL‐12/IL‐23 inhibitor	[227]
	Spesolimab	IL‐36R	IL‐36 receptor inhibitor, monoclonal antibody	[167]

***Advanced therapeutics in AD*** including biologic agents and small molecule inhibitors.

### 3.2 Advanced therapeutics in AD including biologic agents and small molecule inhibitors

Biologic agents allow specific targeting of molecules, which can be further upstream in inflammatory pathways. As discussed above, targeting key proteins in the initiation (TSLP, IL‐18), effector phase (IL‐4, IL‐13) or the crosstalk to neurons (IL‐31) may interfere with the innate‐adaptive interaction, relieve symptoms and restore barrier integrity. Some have been used in Dermatology more than two decades for other inflammatory cutaneous disorders^141^ and especially to great effect for treating psoriasis. However the development of this line of therapeutics for AD has been slower.^142^ Reasons for this slower development for AD include the complex heterogeneity of acute and chronic inflammation seen in AD, the multiple as yet poorly characterised endotypes and the multifactorial causes and exacerbators of AD, including bacterial, viral and fungal dysbiosis.

***TSLP*:** Proinflammatory stimuli generate TSLP. T_H_2 cytokine production by DCs is induced by TSLP, which is upstream from IL‐4, ‐5 and ‐13. Tezepelumab is a human IgG monoclonal antibody that binds TSLP and stops further interactions with the receptor complex. A phase 2b, clinical study showed that there was a trend of improvements in clinical scorings for patients treated with tezepelumab and topical corticosteroid versus placebo; however, significance was not reached.^143^ Considering that TSLP is a driver of one of the pathways that innate immunity influences the downstream inflammation that occurs in AD and other parts of the atopic march such as asthma and allergic rhinitis, further investigations may demonstrate how influencing the inflammatory pathway at an earlier stage can reduce the range of severity of acute and chronic atopic inflammation.

***PDE4:*** Studies have shown that phosphodiesterase (PDE) inhibitors altered inflammatory pathways stimulated in AD and could be considered as a therapeutic target for a non‐steroid based topical treatment for AD.^144^, ^145^, ^146^ Crisaborale is a topical PDE4 inhibitor which has been authorised for the treatment of atopic dermatitis in the US and the European Union. Proinflammatory cytokine responses arise from the conversion by PDE4 of intracellular messenger cyclic adenosine monophosphate (cAMP), into adenosine monophosphate. In AD there is up‐regulation of PDE4 with over‐expression of cytokines such as IL‐4, ‐13, ‐31, released by both innate and adaptive immune cells.^145^

***IL‐4/IL‐13:*** Dupilumab, now licenced for AD treatment in the United States, Europe, China and Japan is a fully human anti‐IL‐4 receptor alpha monoclonal antibody, which inhibits IL‐4 and IL‐13.^147^ Both cytokines are potently produced by cells of the innate immune system and act on innate and adaptive immune cells. Thus the cell‐specific contribution of IL‐4RA‐blockade remains to be determined. Considerable decreases in clinical scores and pruritus of AD were seen, and no systemic side effects were noted, but conjunctivitis incidence was increased.^148^ Importantly, dupilumab therapy reduced type 2 inflammation, reversed AD‐induced epidermal abnormalities and increased gene expression of barrier‐associated proteins.^126^, ^149^

***IL‐13:*** A clinical trial by Wollenberg et al. showed that inhibiting IL‐13 in adults with moderate to severe AD, led to significant clinical improvements in Eczema Area and Severity Index (EASI) and Dermatology Life Quality Index scoring.^150^ Tralokinumab is a fully human IgG4 monoclonal antibody, which targets IL‐13 and was administered in a phase 2b randomised study with concomitant topical glucocorticoids. The safety profile in this trial was in line with those of previous trials of tralokinumab in patients with asthma. None of the adverse events reported were associated with the study drug.^150^ Similarly, lebrikizumab, developed for the treatment of asthma, selectively targets IL‐13 and appears to be effective in moderate‐to‐severe AD.^151^, ^152^ Because ILC2 are implicated in AD pathogenesis and can produce large amounts of IL‐13, future research should include the identification of strategies to interfere with ILC2 function.

***IL‐18:*** IL‐18 contributes to the change from an acute T_H_2‐driven AD endotype towards T_H_1 polarization in chronic disease.^153^ The receptor for IL‐18 signals via the innate inflammatory MyD88‐pathway and can activate T_H_1 cells, basophils, NK cells, mast cells.^154^ Hu et al. reported that serological IL‐18 and IL‐18 binding protein was found in increased amounts—specially during worsening pathology—n patients with AD. In a murine model of eczema increased mast cells in lesional skin and elevated levels of IL‐18BP^+^ mast cells in lesional skin were found.^154^ Allergen challenge resulted in amplified expression of IL‐18, IL‐18BP and IL18 R mRNA. This study suggested that IL‐18 inhibitor agents may be a therapeutic option for AD.^154^

***IL‐31:*** IL‐31 is associated with pruritus that occurs with AD and plays a pathogenic role in the progress of inflammation. Nemolizumab is an IL‐31RA humanised monoclonal antibody which blocks the effects of IL‐31.^155^ Ruzicka et al. highlighted the potential for looking at the IL‐31 pathway as treatment option in AD when significant improvements were seen in pruritus scoring in a phase 2, randomised, double‐blind, placebo‐controlled clinical trial. No specific safety signals were noted however the authors discuss that limited size of a trial precludes clear conclusions about potential side effects of nemolizumab.^155^ Thus, interference with the immune cell‐neuron‐crosstalk may be an important step to break the itch‐scratch‐inflammation cycle and restore the physical skin barrier.

***IL‐33:*** IL‐33 is an inflammatory cytokine associated with innate immunity and can activate ILC2s.^156^ IL‐33 is overexpressed in keratinocytes of AD patients.^157^, ^158^ The activation of ILC2s may contribute to IL‐33‐driven AD‐like inflammation in mice with increases in IL‐5 and IL‐13.^156^ Initial studies on the efficacy of etokimab targeting IL‐33 in human moderate‐to‐severe AD showed moderate but sustained improvement in disease severity.^159^ While this improvement is associated with reducing innate inflammatory pathways by inhibiting IL‐33, basophils can also activate ILC2s induced by IL‐33, which work via IL‐4 in AD‐like inflammation in mouse models.^119^ IL‐33 has been shown to decrease the expression of filaggrin in the stratum corneum, which decreases the barrier function of the epidermis.^116^ Markers that are only expressed on human ILC2s have not been established. By inhibiting IL‐33 the impact on AD of innate and acquired immunity can be modified.

***JAK‐STAT:*** The JAK‐STAT pathway mediates translation of cytokine stimulation into cellular effector function. Recent studies have delivered evidence for the use of JAK inhibitors in treating alopecia areata, psoriasis, vitiligo and AD.^160^, ^161^

Both topical and oral formulations of JAK inhibitors have been assessed in clinical trials. Topical tofacitinib—targeting JAK1 and JAK3, which are downstream of the receptors for IL‐2, IL‐7, IL‐4, IL‐13, IL‐6, IL‐21, type I and II interferons, and others—was shown to bring about a reduction in EASI versus placebo at week 4 (*p* < 0.001) as well as significant results in physician global assessment (PGA), body surface area (BSA) and pruritus scores.^162^ A pilot study of oral tofacitinib showed a decrease in the scoring of AD during treatment for AD.^163^

The nature of broad inhibition of cytokine signalling increases the risk of infections as innate and adaptive immune responses are effectively inhibited. Indeed, infections of the upper respiratory tract are among the most common adverse events. Whether inhibition of JAKs will improve barrier function remains to be determined.

### 3.3 Advanced therapeutics in psoriasis

***TNF:*** Etanercept (TNF inhibitor), infliximab (chimaeric TNF‐neutralising antibody), adalimumab (anti‐TNFα), certolizumab pegol (pegylated anti‐TNF‐Fab fragment) interfere with the actions of TNF during moderate‐to‐severe psoriasis. As TNF downregulates filaggrin and loricrin, inhibition of TNF can help to restore skin barrier integrity.^164^

***PDE4:*** As discussed above, inhibiting PDE4 works in an anti‐inflammatory action rather than immunosuppressant activity. Proinflammatory cytokines are decreased allowing greater expression of anti‐inflammatory mediators by intracellular inhibition of cAMP degradation and increased levels of cAMP at the intracellular level.^165^

***IL‐12/IL‐23:*** Ustekinumab binds the p40‐subunit shared by IL‐12 and IL‐23 and inhibits binding to their receptors, thus interfering with the polarization of T cells by innate immunity but increasing the risk of infections as innate immune cells cannot polarise T cells towards T_H_1. Guselkumab, tildrakizumab and risankizumab bind the p19‐subunit of IL‐23 and prevents the T_H_17‐polarization of T cells, and subsequently epidermal hyperproliferation, keratinocyte activation and inflammation.

***IL‐17:*** Interference with the IL‐17‐mediated inflammation is achieved by treatment with secukinumab (anti‐IL‐17A), ixekizumab (anti‐IL‐17A), and brodalumab (anti‐IL‐17RA). As IL‐17 can downregulate filaggrin expression in keratinocytes,^166^ inhibition of the IL‐17‐pathway alleviates psoriatic symptoms and facilitates skin barrier restoration.

***IL‐36R:*** Targeting the IL‐36 pathway may pose a novel treatment option for generalized pustular psoriasis.^167^ A single dose of BI655130/Spesolimab improved skin symptoms in study participants within two weeks.^167^ Mechanistically, IL‐36R expressed by IL‐17A‐activated keratinocytes may be blocked by Spesolimab, which may break the pro‐inflammatory cycle during chronic psoriasis and GPP.^168^

### 3.4 Gene polymorphisms and prediction of response to biologicals and other therapies

Similar to the role of gene polymorphisms with regard to disease susceptibility, these polymorphisms also play an important role in the response to therapy. Two extensive reviews of how and which gene polymorphisms influence the efficiency of different therapies for psoriasis have been published by Linares‐Pineda et al.^169^ and more recently by Membrive Jiménez et al.^170^ Additionally, Prieto‐Perez and co‐workers reviewed anti‐TNF‐treatment efficacy in psoriasis with a focus on related autoimmune disorders, such as rheumatoid arthritis.^171^

The majority of the described gene polymorphisms are in relation to TNF and its intracellular signalling, which is probably attributable to the fact that anti‐TNF treatments were first approved and the most prescribed biologic treatments for psoriasis. Even though there are some studies on the association of gene polymorphisms with treatment response to other biologicals (e.g. IL‐12/23 inhibitor ustekinumab), this field requires more in‐depth research to clearly elucidate the roles of different SNPs on the efficacy of advanced treatment options. Currently, there is a lack of research on the influence of SNPs in treatments for diseases other than psoriasis and thus limiting the potential of gene polymorphisms as useful biomarkers in personalised medicine.

## 4 CONCLUSION

The global research effort to advance our understanding of inflammatory skin diseases, such as psoriasis and AD, led to the recognition of the importance of immunological processes underlying pathogenesis (Figure 1). The realisation that skin barrier integrity can be modified by immunological factors unlocked a new perspective on the genesis of AD. Thus, restoration of an intact functional skin barrier needs to be one of the main objectives in successfully ameliorating AD. The advent of novel treatments targeting single components or shared pathways involved in genesis of skin inflammation will allow us to tailor therapies to the patient across the AD to psoriasis disease spectrum.

## CONFLICT OF INTEREST

None to declare.

## AUTHOR CONTRIBUTIONS

**Heike C. Hawerkamp:** Investigation‐Equal, Writing – original draft‐Equal, Writing – review & editing‐Equal; **Caoimhe M. R. Fahy:** Investigation‐Supporting, Writing – original draft‐Equal, Writing – review & editing‐Equal; **Padraic G. Fallon:** Conceptualization‐Equal, Investigation‐Equal, Supervision‐Supporting, Writing – original draft‐Equal, Writing – review & editing‐Supporting; **Christian Schwartz:** Conceptualization‐Equal, Investigation‐Equal, Supervision‐Lead, Visualization‐Lead, Writing – original draft‐Equal, Writing – review & editing‐Equal.

## Data Availability

Data sharing not applicable to this article as no datasets were generated or analysed during the current study.
